# Peptide Biomarkers Discovery for Seven Species of Deer Antler Using LC-MS/MS and Label-Free Approach

**DOI:** 10.3390/molecules27154756

**Published:** 2022-07-25

**Authors:** Fei Xue, Bing Wang, Dong-Xiao Guo, Yang Jiao, Xue Yin, Wei-Liang Cui, Qian-Qian Zhou, Feng-Rui Yu, Yong-Qiang Lin

**Affiliations:** NMPA Key Shandong Engineering Laboratory for Standard Innovation and Quality Evaluation of TCM, Shandong Engineering Research Center for Generic Technologies of Traditional Chinese Medicine Formula Granules, Laboratory for Quality Evaluation of Gelatin Products, Shandong Institute for Food and Drug Control, Jinan 250101, China; xuefei199011@hotmail.com (F.X.); sdifdczytg1@163.com (B.W.); sdifdczytg2@163.com (D.-X.G.); sdifdczytg3@163.com (Y.J.); sdifdczytg4@163.com (X.Y.); sdifdczytg5@163.com (W.-L.C.); sdifdczytg6@163.com (Q.-Q.Z.); sdifdczytg7@163.com (F.-R.Y.)

**Keywords:** adult beta-globin, deer antler, peptide biomarkers, deer species identification, label-free approach

## Abstract

Deer antler is a globally widely used precious natural medicine and the material of deer horn gelatin. However, identification of deer antler species based on traditional approaches are problematic because of their similarity in appearance and physical-chemical properties. In this study, we performed a comprehensive antler peptidome analysis using a label-free approach: nano LC-Orbitrap MS was applied to discover peptide biomarkers in deer adult beta-globin (HBB_A_), and HPLC-Triple Quadrupole MS was used to verify their specificity. Nineteen peptide biomarkers were found, on which foundation a strategy for antlers and a strategy for antler mixtures such as flakes or powder are provided to identify seven species of deer antler including Eurasian elk (*Alces alces*), reindeer (*Rangifer tarandus*), white-tailed deer (*Odocoileus viginianus*), white-lipped deer (*Przewalskium albirostris*), fallow deer (*Dama dama*), sika deer (*Cervus nippon*), and red deer (*Cervus elaphus*) simultaneously. It is worth noting that our search found that the HBB_A_ gene of sika deer, red deer, and North American wapiti (*Cervus canadensis*) in China may have undergone severe genetic drifts.

## 1. Introduction

Deer antler is the only completely regenerable organ found in mammals, which can also be used as a crucial natural medicine [1,2]. The earliest records of medicinal use of deer antler date back 2000 years [3]. In recent years, people have studied the chemical composition, bioactive components, and pharmacological effects of deer antler [2,4], which has been proved to be an important nourishing and health care product with high nutritional and medicinal value; it is rich in protein [5], and has certain curative effects against breast cancer [6], prostate cancer [7], acute kidney injury [8], physical decline [9], sexual dysfunction [10], neuronal damage [11], cardiac fibrosis [12], ischemic heart disease [13], vascular disease [14], osteoporosis [9], hepatocyte injury [15], and so on. Deer antler is also the raw material of deer horn gelatin [16], which has been proven to improve blood circulation. On account of its multi-functions, a huge supply and demand of deer antler has been created, with the world producing more than 1000 tons of deer antlers per year. New Zealand, China, Russia, America, and Canada produce about 450, 400, 80, 20, and 20 tons of deer antler each year, respectively [17].

At present, the health care function of deer antler is recognized worldwide; however, due to the influence of natural medicine medical theories and differences in historical and cultural heritage, different species of deer antler have huge price differences in different regions. For example, in China, the antler of sika deer (*Cervus nippon*) and red deer (*Cervus elaphus*) are authentic species with the highest prices [18]. The price of sika deer antler in the Chinese market is 10 times of that of reindeer (*Rangifer tarandus*) antler, and the phenomenon of selling reindeer antler as red deer antler or sika deer antler often appears under the temptation of high profits. Besides, Eurasian elk (*Alces alces*) antlers are also used to pretend to be red deer antler and sika deer antler in deer horn gelatin production. The white-lipped deer (*Przewalskium albirostris*) is an endangered species in China [19], making its antler extremely precious. The white-tailed deer (*Odocoileus viginianus*) are widely distributed in North America [20,21,22], and fallow deer (*Dama dama*) are found in southern Europe [23,24,25]. The antlers from the white-tailed deer and fallow deer are mostly used for exportation. Because all these seven species of deer antlers have different value, but it is hard to tell them apart because of their similarity, they have become the research objects of our experiment.

Based on the fact that the price is expensive, it is mostly to sell deer antler as a mixture such as flakes and powder. Therefore, the risk of imitating or tampering with the high-priced antler with a low-priced one is very high. For the situation that the species of deer antlers is confusing and difficult to identify, it is very difficult to distinguish the species in traditional approaches (e.g., microscopic identification and physical and chemical identification), and a method with strong specificity and high detection efficiency is urgently needed to solve this problem. Researching and developing the method to distinguish deer antler of different species at the proteomic level is meaningful.

Peptide biomarkers are a unique and stable segment of a certain species [26]. By using LC-MS/MS technology, it can be determined whether there is the specie by confirming the presence or absence of this segment of peptides. Adult beta-globin (HBB_A_) is found in the red blood cells of non-infant animals, with a total length of 145 amino acids, which can be detected as long as there is a trace amount of blood [27]. We found that it exists in high abundance in deer antler, so it is believed that HBB_A_ is the key to provide a source of peptide biomarkers.

**Figure 1:**
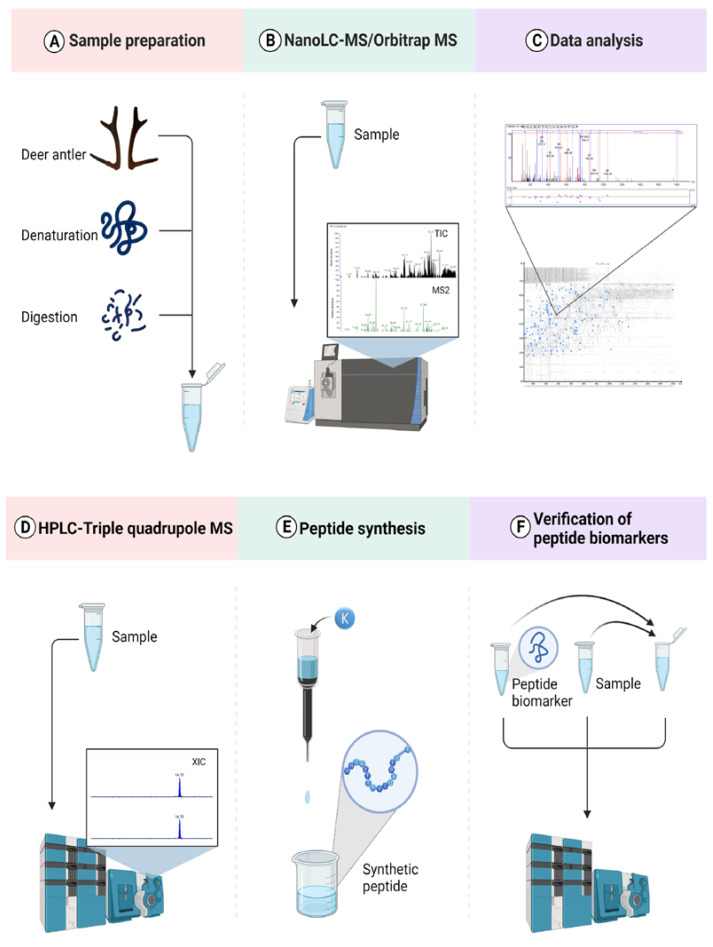
Analytical scheme for identifying peptide biomarkers in deer antlers. (Adapted from “Snapshot Metabolomics”, by BioRender.com (2022). Retrieved from https://app.biorender.com/biorender-templates (accessed on 25 May 2022)). (**A**) Deer antlers were denatured by guanidine hydrochloride and enzymatic hydrolysis by trypsin. (**B**) Analysis of relative deer antler enzymatic hydrolysate was conducted by Nano LC-Orbitrap MS. (**C**) Mass spectrometry data were used in the PEAKS software for analysis, followed by de novo sequencing of peptides, alignment in the database, and generation of peptides biomarkers. (**D**) HPLC-Triple Quadrupole MS analyzed seven species of deer antler enzymatic products in MRM mode. (**E**) Purified peptide biomarkers were obtained by chemical synthesis. (**F**) HPLC-Triple Quadrupole MS was used to confirm the sequence of peptide biomarkers.

In this study, we performed a comprehensive antler peptidome analysis using a label-free approach; peptide biomarkers in deer’s HBB_A_ were found by nano LC-Orbitrap MS combined with data analysis software, and HPLC-Triple Quadrupole MS was used to verify the specificity of peptide biomarkers and establish the identification method. A total of 19 peptide biomarkers were found, which can successfully distinguish the antlers of seven species of deer. Furthermore, a strategy to identify seven species of deer antlers simultaneously and a strategy to identify deer antler mixtures such as flakes or powder simultaneously were provided as well. A flowchart of our experimental design is shown in Figure 1.

## 2. Results and Discussion

### 2.1. Results of Nano LC-MS/MS Data Analysis

Nano LC-Orbitrap MS analyzed the peptide mixture produced after processing the antler samples. High-resolution mass spectrometry data of all the antler protein enzymolysis peptides were obtained and imported into PEAKS software for analysis. A total of 33,185 peptides were sequenced de novo, which were identical to the “adult beta-globin of deer” database, and a total of 333 peptides were identified. The specific results of the analysis are shown in Table S3 and Figure S1 (See Supplementary Materials).

### 2.2. Determination and Specificity Verification of Peptide Biomarkers

**Figure 2:**
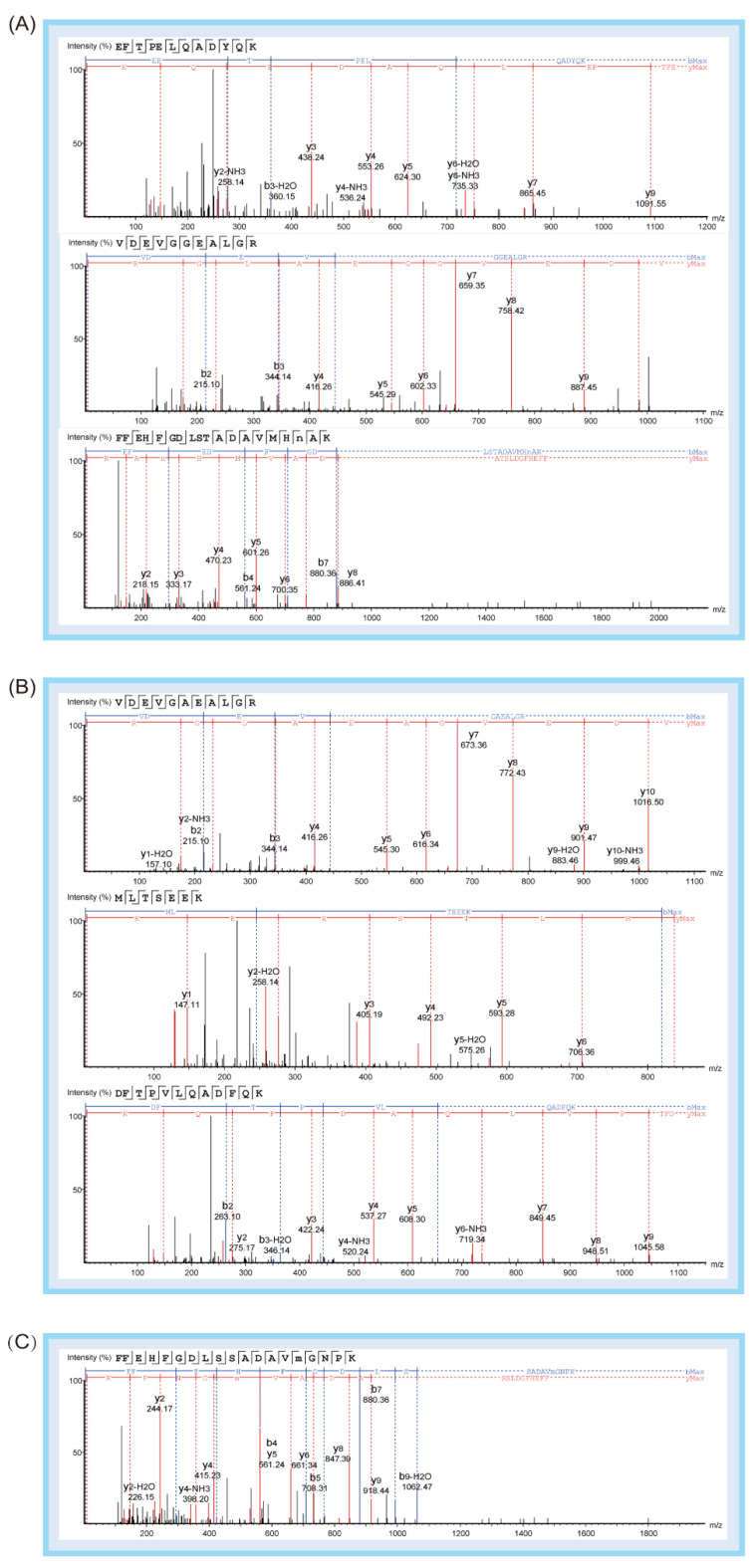

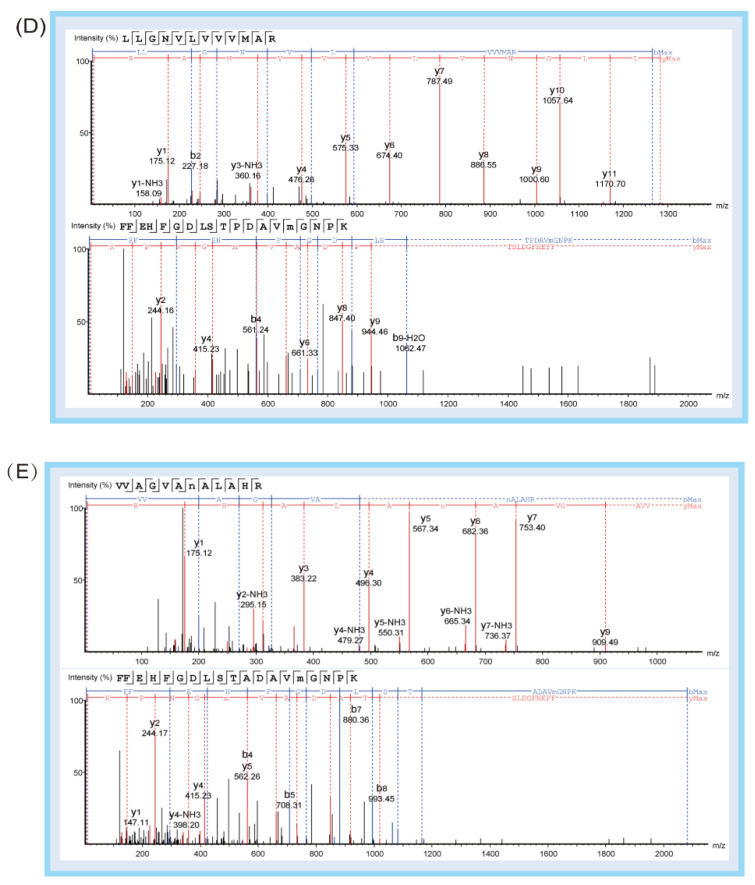
Corresponding sequence analysis maps (b-blue, y-red) of 11 peptide biomarkers relevant to the identification strategy 1. (**A**) This graph shows three Eurasian elk-specific peptide biomarkers: Peptide 1 (EFTPELQADYQK), Peptide 2 (VDEVGGEALGR), and Peptide 3 (FFEHFGDLSTADAVMHNAK). (**B**) This graph shows three reindeer-specific peptide biomarkers: Peptide 4 (VDEVGAEALGR), Peptide 5 (MLTSEEK), and Peptide 6 (DFTPVLQADFQK). (**C**) This graph shows one white-tailed deer-specific peptide biomarker: Peptide 7 (FFEHFGDLSSADAVMGNPK). (**D**) This graph shows two white-lipped deer-specific peptide biomarkers: Peptide 8 (LLGNVLVVVMAR) and Peptide 9 (FFEHFGDLSTPDAVMGNPK). (**E**) This graph shows two sika deer-specific peptide biomarkers: Peptide 10 (VVAGVANALAHR) and Peptide 11 (FFEHFGDLSTADAVMGNPK).

**Figure 3:**
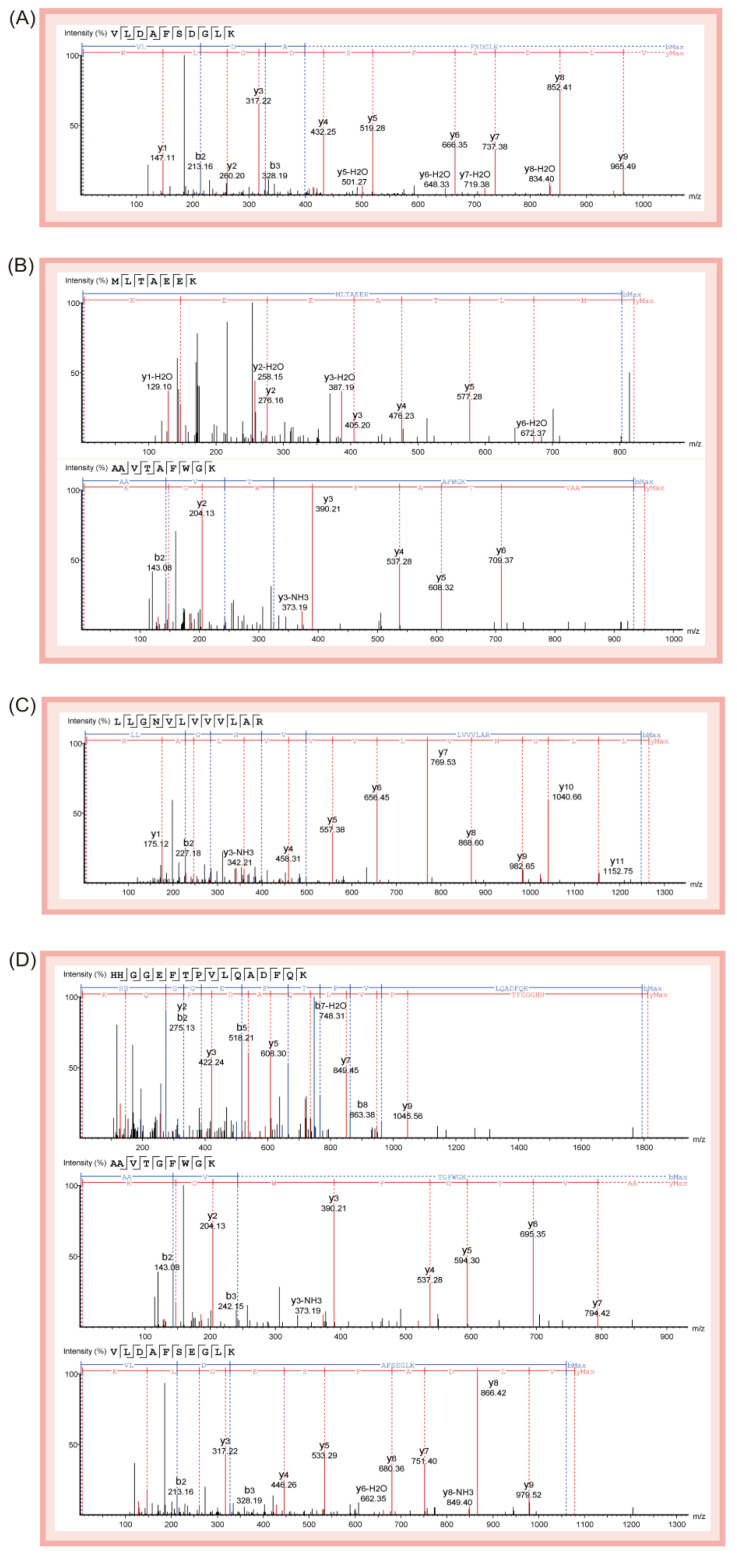

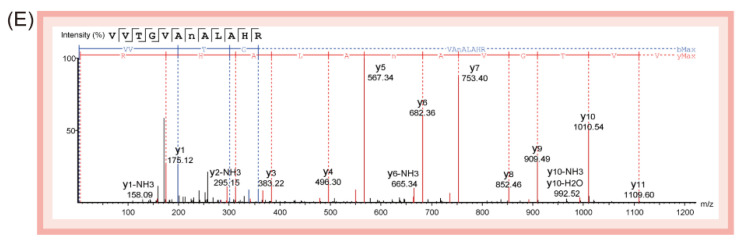
Corresponding sequence analysis maps (b-blue, y-red) of eight peptide biomarkers relevant to identification strategy 2. (**A**) This graph shows one common peptide biomarker except Eurasian elk: Peptide 12 (VLDAFSDGLK). (**B**) This graph shows two common peptide biomarkers except reindeer: Peptide 13 (MLTAEEK), Peptide 14 (AAVTAFWGK). (**C**) This graph shows one common peptide biomarker except white-lipped deer: Peptide 15 (LLGNVLVVVLAR). (**D**) This graph shows three common peptide biomarkers except fallow deer: Peptide 16 (HHGGEFTPVLQADFQK), Peptide 17 (AAVTGFWGK), and Peptide 18 (VLDAFSEGLK). (**E**) This graph shows one common peptide biomarker except sika deer: Peptide 19 (VVTGVANALAHR).

To verify the specificity of peptide biomarkers, the protein identification results of PEAKS software were investigated. In previous experiments, we found that HBB_A_ was a highly abundant protein in deer antler. The amino acid sequence of HBB_A_ is highly conserved; we believe that it is significant for the discovery of deer antler peptide biomarkers and the identification of antler species. Therefore, the “adult beta-globin of deer” database was selected to analyze the mass spectral data obtained by Nano LC-Orbitrap MS. To find peptide biomarkers, we used HPLC-Triple Quadrupole MS to verify the specificity of the 333 identified peptides. MRM is one of the most commonly used mass spectrometry-based targeted analysis methods that can focus on detecting peptides of interest with high sensitivity. Because the analysis method can be controlled in about 20 min, and the instrument is of high-cost performance and easy to operate, it is widely used in the world inspection industry. The specificity of peptides in the HHB_A_ identified by PEAKS software was verified, and the parent ions and product ions with the highest response intensity were investigated and selected as qualitative ions. With three different C18 columns, this method was validated to be capable to identify the peptide biomarkers. As is shown in the results, 19 peptide biomarkers were found, each with different specificity (Table 1). The corresponding sequence analysis maps of peptide biomarkers are shown in Figure 2 and Figure 3.

### 2.3. Synthesis and Sequence Verification of Peptide Biomarkers

To increase the reliability of the study, peptide biomarkers were synthesized and analyzed by HPLC-Triple Quadrupole MS. The results showed that the peak time of synthetic peptides was consistent with the peak time of samples. The synthetic peptides were then added to seven species of deer antler samples for analysis. The results showed that the peak time of peptide-added samples was the same as that of antler samples, with only one peak. Therefore, the synthetic sequence is considered to have the same amino acid sequence as the peptide biomarkers.

### 2.4. Strategy 1: Simultaneous Identification of Seven Species of Deer Antlers

**Figure 4:**
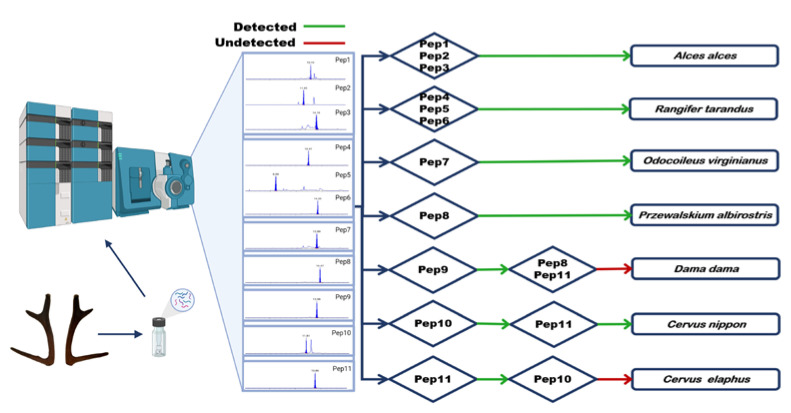
Strategy 1: Simultaneously identifying seven species of deer antler. The deer antlers were processed by method 3.2 and then analyzed by HPLC-Triple Quadrupole MS. The peptide biomarkers in the diamond box were used to determine the species; red line means undetected, green line means detected.

Based on the peptide biomarkers discovered, a strategy was established to simultaneously identify seven species of deer antlers using the MRM mode for intact antler samples. The digested antlers peptide mixture was analyzed by HPLC-triple quadrupole MS. According to the peptide biomarkers in the diamond box of the strategy flow chart (Figure 4), seven species of deer antlers were simultaneously identified. This strategy shows excellent specificity and covers the common deer antler varieties in the East Asian market. It is of specific significance for regulating the deer antler market and guiding the correct use of deer antler varieties.

### 2.5. Strategy 2: Identification of Mixtures Such as Deer Antler Flakes or Deer Antler Powder

**Figure 5:**
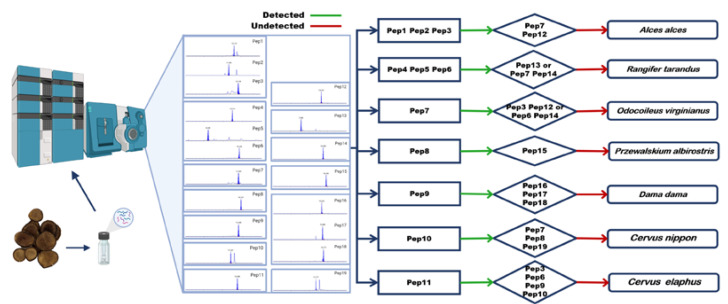
Strategy 2: Simultaneously identifying seven species of deer antler flakes and powder. The deer antler flakes and powder were processed by method 3.2 and then analyzed by HPLC-Triple Quadrupole MS. The peptide biomarkers in the rectangular box were used to preliminarily identify species, and peptide biomarkers in diamond box were used to further identification and checking for adulteration; red line means undetected, green line means detected.

The high price of deer antler causes antler flakes and antler powder to be the most widely circulated products, making it more susceptible to adulteration due to loss of entire appearance. In response to this situation, a strategy was established to simultaneously identify the species of antler flakes or antler powder and check for adulteration. The process is divided into identifying species (rectangular box) and checking for adulteration (diamond box). HPLC-Triple Quadrupole MS is used to analyze the peptide mixture after enzymatic digestion of the deer antler sample. Whether the antler mixture sample is from a single species can be identified according to the strategy flow chart (Figure 5).

### 2.6. Reasons for Choosing HBB_A_

Deer antler has a high-speed growth rate and a well-developed vascular network [28,29,30] while the blood is rich in protein [31], so the differences between different deer antlers can be found through the differences in peptides of blood proteins. Moreover, the sequence of HBB_A_ is highly conserved, which means that the amino acid fragments in the protein remain unchanged during evolution [27]. Therefore, it can be used as a research object for the identification method. The HBB_A_ of deer is a protein with a sequence of 145 amino acids, which is divided into three segments when encoded by genes, 1–29, 30–103, and 104–145.

### 2.7. Amino Acid Sequence of HBB_A_ in Deer Antler

#### 2.7.1. Amino Acid Sequence of HBB_A_ in Sika Deer Antler

**Figure 6:**
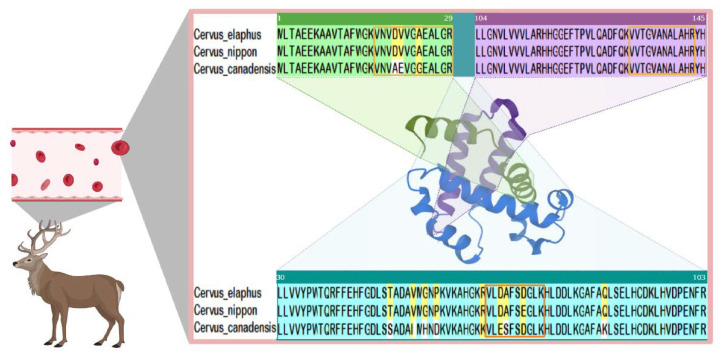
Sequence alignment of deer’s HBB_A._ (Adapted from “Blood Oxygenation”, by BioRender.com (accessed on 25 May 2022. Retrieved from https://app.biorender.com/biorender-templates (accessed on 25 May 2022). Structure of HBB_A_ downloaded from AlphaFold Protein Structure Database [32,33], Hemoglobin subunit beta-A, Source organism: *Bos javanicus*, https://www.alphafold.ebi.ac.uk/entry/P04346 (accessed on 23 May 2022). The HBB_A_ amino acid sequences were downloaded from UniPort (http://www.uniprot.org/ (accessed on 2 March 2022)). The green part represents amino acids 1–29, the blue part represents amino acids 30–103, and the purple part represents amino acids 104–145. Yellow labels represent amino acids with a similarity greater than 50%.

Despite that most peptide biomarkers are consistent with the amino acid sequences provided on Uniport, there are still subtle differences in individual deer species. For example, the database sequence of Pep 10 in sika deer antler should be VVTGVANALAHR, which was found to be VVAGVANALAHR in our experiment; this sequence exists in the part of the 104–145 segment of HBB_A_ (the purple part is marked with an orange box in Figure 6).

#### 2.7.2. Amino Acid Sequence of HBB_A_ in Sika Deer, Red Deer and North American Wapiti Deer Antler

Moreover, Pep 18, which should be detected in sika deer antler, was not detected, while Pep 12 with the sequence VLDAFSDGLK was detected instead. We also detected the peptide sequence VLESFSDGLK, which is theoretically exclusive to North American wapiti, but detected in some red deer and sika deer, come from in 30–103 (the blue part is marked with an orange box in Figure 6). It is the same for peptides of the sequence VNVAEVGGEALGR, from 1–29 (the blue part is marked with an orange box in Figure 6). Besides, two peptides described above, Pep 11 and Pep 16, exclusive to red deer and sika deer, were also detected in our North American wapiti antler samples.

It is worth noting that red deer, sika deer, and North American wapiti are extensively used in traditional Chinese medicine as the authentic deer antler source (North American wapiti is used as a subspecies of red deer). For thousands of years, deer have been an important domestic economy animal in China, the antler weight trait has been a single breeding goal, and strong artificial selection may have led to severe genetic drift in the breeding populations of red deer, sika deer, and North American wapiti. Our laboratory continues to collect samples of North American wapiti antlers for further analytical validation.

### 2.8. Post-Translational Modifications (PTM) of Peptide Biomarkers

**Figure 7:**
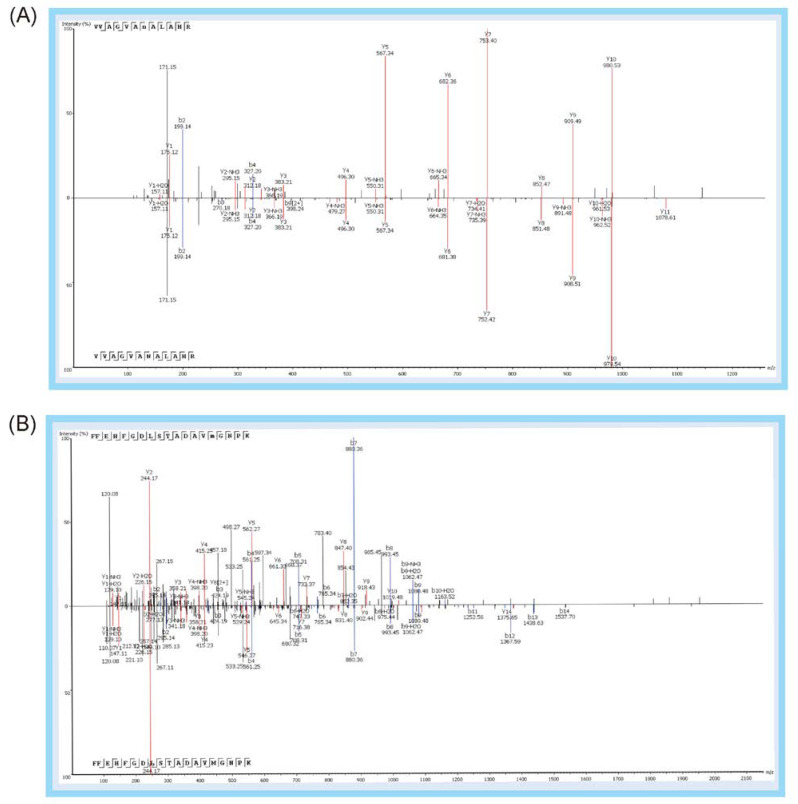

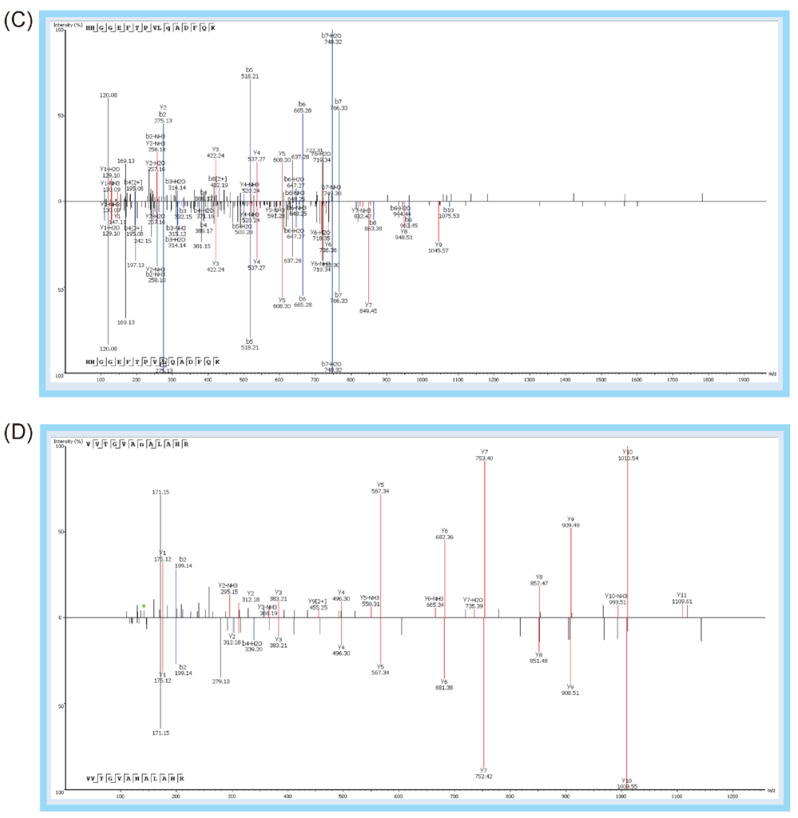
Comparison of sequence analysis maps after and before PTM of Pep 10 (**A**), Pep 11 (**B**), Pep 16 (**C**), and Pep 19 (**D**).

In the experiment, it was found that the most common post-translational modifications (PTM) of peptide biomarkers were the deamidation modification of asparagine and glutamine, which increased the molecular weight of the peptide by 0.98 Da, and the oxidative modification of methionine, which increased the molecular weight of the peptide by 15.99 Da (Table 2). It was indicated that Pep 11 FFEHFGDLSTADAVMGNPK is highly specific after methionine oxidative modifications and can be utilized as a unique inspection peptide for red deer and sika deer. This is of great significance to China, where red deer and sika deer serve as the species of genuine antlers. The same methionine modification occurs in Pep 7 and Pep 9. Both pre- and post-modification peptides of Pep 10, Pep 11, Pep 16, and Pep 19 were detected in samples (Figure 7), while Pep 3, Pep 7, and Pep 9 could only be detected in the post-modified peptides samples.

## 3. Materials and Methods

### 3.1. Materials and Reagents

Trypsin (proteomics grade), guanidine hydrochloride, hydroxymethyl aminomethane (Tris), ethylenediaminetetraacetic acid (EDTA), ammonium bicarbonate, and acetic acid (biotech grade) were purchased from Sigma-Aldrich (St. Louis, MO, USA), formic acid (optima LCMS) was obtained from Thermo Fisher Scientific (Waltham, MA, USA), acetonitrile (gradient grade) and methanol (gradient grade) was purchased from Merck KGaA (Darmstadt, Germany). Ultrafiltration centrifuge tubes (3 kDa, 0.5 mL) were purchased from Merck Millipore (Burlington, MA, USA). Water was prepared on a Milli-Q A 10 Gradient system by Millipore (Schwalbach, Germany).

Twenty-three batches of deer antlers were collected from markets in different regions of China. The species of deer antlers were identified by the Shandong Academy of Agricultural Sciences using the DNA barcode method; 3 batches of reindeer antlers were identified by the method provided by literature [3], and the sample information is shown in Table S1. Seven species of deer antlers were used to discover peptide biomarkers, and all samples were used for validation. The peptide biomarkers shown in Table 1 were chemically synthesized by GL Biochem (Shanghai, China), Yuan Peptide (Nanjing, China) and China peptides (Suzhou, China).

### 3.2. Sample Preparation

Deer antler was sliced and crushed, 50 mg of antler powder was weighed (100 mg for those with a higher degree of ossification), and 10 mL of denaturing buffer (6 M guanidine hydrochloride, 1 M Tris, 2.5 mM EDTA, hydrochloric acid to adjust pH to 8.0) was added. The samples were retained at 80 °C overnight, then cooled down to room temperature. Centrifugalize at 12,000 r for 10 min, discard the lower layer solution, add 500 μL of water, repeat the above operation twice, add 500 μL of 1% Ammonium bicarbonate solution and 20 μL of bovine trypsin solution (10 mg/mL), perform enzymolysis at 37 °C for 15 min, then terminate the enzymatic hydrolysis reaction at 100 °C, let it cool down to room temperature, centrifugalize at 12,000 r for 10 min, and take the supernatant. The resulting product was used for Nano LC-MS/MS analysis.

### 3.3. Nano LC-MS/MS Analysis

The prepared samples of deer antlers were analyzed by nanoliter liquid phase (EASY-nLC 1000, Thermo Scientific, San Jose, CA, USA) coupled with high-resolution mass spectrometry (Orbitrap-Fusion, Thermo Scientific, San Jose, CA, USA). Desalting and enrichment were performed by a C18 column (100 μm × 3.5 cm, 5 μm, Thermo Scientific, San Jose, CA, USA), separation was by a C18 column (75 μm × 15 cm, 3 μm, Thermo Scientific, San Jose, CA, USA), flow rate was 300 nL/min, mobile phase A was 0.1% (*v/v*) formic acid aqueous solution containing 2% acetonitrile, and mobile phase B was 0.1% (*v/v*) formic acid aqueous solution containing 98% acetonitrile. Gradient elution was performed as follows (0~1 min, 1%B→6%B, 1~96 min, 6%B→22%B, 96~113 min, 22%B→30%B, 113~117 min, 30%B→95%B, 117~120 min, 95%B). The injection volume was 1 μL, content of 200 ng peptides.

The high-resolution mass spectrometry parameters were set as follows: ion source was Nanospray Flex, analysis was performed in positive ion mode, spray voltage was 1800 V, ion transmission capillary temperature was 275 °C, and S-Lens transmission efficiency was set to 60%. The primary mass spectrometer used Orbitrap as the mass analyzer with a resolution of 60,000, and acquisition range was 350~1550 (*m/z*); the secondary mass spectrometer used Orbitrap as the mass analyzer, and the rapid scan mode was used for scanning, top 20 data-dependent mode was applied, precursor ion selection was performed, and fragmentation was performed in HCD mode with the fragmentation energy NCE set to 30%.

### 3.4. Mass Spectrometry Data Analysis and Discovery of Peptide Biomarkers

The resulting MS/MS data were analyzed using PEAKS Studio Software (8.5 Edition, Bioinformatics Solutions Inc., Waterloo, ON, Canada). The setting parameters were as follows: query type was homology match, variable modifications were Carbamidomethylation: 57.02, Oxidation (M): 15.99, Acetylation (K): 42.01, parent mass error tolerance was 15.0 ppm, fragment mass error tolerance was 0.02 Da, max missed cleavages was 3, max variable PTM per peptide was 6, de novo score (%) threshold was 15, filter charge was 2–8, de novo sequencing and peptide matching of all peptides were performed using the databases of “adult beta-globin of deer” (Contains a total of 15 HBB_A_ proteins from 15 deer species, download from Uniport, https://www.uniprot.org (accessed on 2 March 2022); for more detailed counting, see Table S2).

### 3.5. Verification of the Specificity of Peptide Biomarkers by HPLC-MS/MS

HPLC-Triple Quadrupole MS (QTRAP6500 LC/MS, AB SCIEX, Foster City, CA, USA) was used to verify specificity and establish the MRM method. The chromatographic column was C18 (2.1 mm × 100 mm, 1.8 μm, ZORBAX SB RRHD, Agilent Technologies, Santa Clara, CA, USA; 2.1 mm × 100 mm, 1.8 μm, ZORBAX Eclipse RRHD, Agilent Technologies, Santa Clara, CA, USA; 2.1 mm × 100 mm, 1.7 μm, ACQUITY UPLC HSS, Waters Corporation, Milford, MA, USA). After exploration, the liquid phase parameters were set as follows: the column temperature was 43 °C, the flow rate was 0.3 mL/min, and the mobile phase A was an aqueous solution containing 0.1% (*v/v*) formic acid, B was an acetonitrile solution containing 0.1% (*v/v*) formic acid, and gradient elution was performed as follows (0~9 min, 3%B→7.5%B, 9~13 min, 7.5%B→25%B, 13~14 min, 25%B→90%B, 14~17 min, 90%B, 17~17.5 min, 90%B→97%B, 17.5~21 min, 97%B). Injection volume was 5 μL, content of 1 μg peptides. The mass spectrometry parameters were set as follows: mode was set as mass detector, electrospray ionization (ESI) and multiple-reaction monitoring in positive ion, sheath gas flow rate was 46 L/h, auxiliary gas flow rate was 850 L/h, spray voltage was 3.5 kV, source temperature was 150 °C, auxiliary gas temperature was 400 °C. Cone voltage was 30 V, and collision voltage was 35 V. The solvent delay was 0~4 min and 16~20 min.

### 3.6. Synthesis and Verification of Peptide Biomarkers

Peptide biomarkers were artificially synthesized and used ultrapure water to make a 10 ng/μL solution. To verify whether the peak times were consistent, HPLC-Triple Quadrupole MS was used on synthesized peptides and deer antler preparation samples. Injection volume was 5 μL (peptides in antler samples injected 1 μg, and synthetic peptides injected 50 ng). The synthesized peptides were added to the deer antler preparation sample with the application of HPLC-Triple Quadrupole MS to verify the number of peaks. Suppose the peak time of the synthetic peptide matches that of the antler sample, and the peptide-added antler sample has only one peak. In that case, it can be assumed that the sequence of the peptide biomarker matches that of the antler sample.

## 4. Conclusions

Deer antler peptide analysis was performed using a label-free method. According to the data analysis results of Nano LC-Orbitrap MS, it is believed that HBB_A_ is the key solution for deer antler identification. Using HPLC-Triple Quadrupole MS to verify the specificity and establish the identification method, a total of 19 peptide biomarkers were found, each with different specificity. A strategy for identifying seven species of deer antler and a strategy for identifying seven species of deer antler flakes or powder were established. It should be noted that the HBB_A_ gene of red deer, sika deer, and North American wapiti in the Chinese range may have suffered severe genetic drift. The two established strategies could identify deer antlers (with more than two prongs) which can be seen in the East Asian market, and would play an important role in regulating the East Asian deer antler market. Moreover, considering that blood is distributed throughout the body of deer, our research has considerable value in identifying deer products such as venison and deer hide. However, the experiment did not identify the theoretical peptide biomarkers of fallow deer. Hence, further research is needed.

## Figures and Tables

**Table 1:** Sequences of peptide biomarkers and their specificity (positive ion mode).

No.	Sequence	Eurasian Elk	Reindeer	White-Tailed Deer	White-Lipped Deer	Fallow Deer	Sika Deer	Red Deer	Charge	*m/z*	Production
Pep 1	EFTPELQADYQK	√							2	734.85	360.15
Pep 2	VDEVGGEALGR	√							2	551.78	659.35
Pep 3	FFEHFGDLSTADAVMHNAK	√							3	713.32	880.36
Pep 4	VDEVGAEALGR		√						2	558.29	772.43
Pep 5	MLTSEEK		√						2	419.20	258.14
Pep 6	DFTPVLQADFQK		√						2	704.86	537.27
Pep 7	FFEHFGDLSSADAVMGNPK			√					3	695.64	880.36
Pep 8	LLGNVLVVVMAR				√				2	642.39	787.49
Pep 9	FFEHFGDLSTPDAVMGNPK				√	√			3	708.99	880.36
Pep 10	VVAGVANALAHR			√	√		√		2	589.83	567.34
Pep 11	FFEHFGDLSTADAVMGNPK						√	√	3	700.32	880.36
Pep 12	VLDAFSDGLK		√		√	√	√	√	2	532.78	317.22
Pep 13	MLTAEEK	√		√	√	√	√	√	2	411.21	258.14
Pep 14	AAVTAFWGK	√			√	√	√	√	2	475.76	390.21
Pep 15	LLGNVLVVVLAR	√	√	√		√	√	√	2	633.42	769.53
Pep 16	HHGGEFTPVLQADFQK				√		√	√	3	604.30	275.13
Pep 17	AAVTGFWGK		√	√					2	468.75	567.34
Pep 18	VLDAFSEGLK	√		√					2	539.79	866.43
Pep 19	VVTGVANALAHR	√	√			√		√	2	604.84	567.34

**Table 2:** Post-translational modifications (PTM) of peptide biomarkers.

No	Sequence	Modification Site	Types of PTM	Change of Molecular Weight	*m/z* Before PTM	*m/z* After PTM
Pep3	FFEHFGDLSTADAVMHNAK	17 N	Deamidation	+0.98	713.00	713.32
Pep7	FFEHFGDLSSADAVMGNPK	15 M	Oxidation	+15.99	690.65	695.64
Pep9	FFEHFGDLSTPDAVMGNPK	15 M	Oxidation	+15.99	703.99	708.99
Pep10	VVAGVANALAHR	7 N	Deamidation	+0.98	589.34	589.83
Pep11	FFEHFGDLSTADAVMGNPK	15 M	Oxidation	+15.99	694.99	700.32
Pep16	HHGGEFTPVLQADFQK	14 Q	Deamidation	+0.98	604.30	604.60
Pep19	VVTGVANALAHR	7 N	Deamidation	+0.98	604.35	604.84

## Data Availability

Not applicable.

## Supplementary Materials

The following supporting information can be downloaded at: https://www.mdpi.com/article/10.3390/molecules27154756/s1, Figure S1: Statistics results of mass spectrometry data analysis; Table S1: Deer antler samples used in this study; Table S2: Database parameters of “adult beta-globin of deer”; Table S3: Statistics results of mass spectrometry data analysis.

## Sample Availability

Not Available.
