# Supplementary material for: Peptide Biomarkers Discovery for Seven Species of Deer Antler Using LC-MS/MS and Label-Free Approach
Source: Molecules. 2022 Jul 25;27(15):4756. doi: 10.3390/molecules27154756 (PMC9331363; doi:10.3390/molecules27154756)
Supplement: Supplementary file 1 [file molecules-27-04756-s001.zip › Figure S1.pdf]

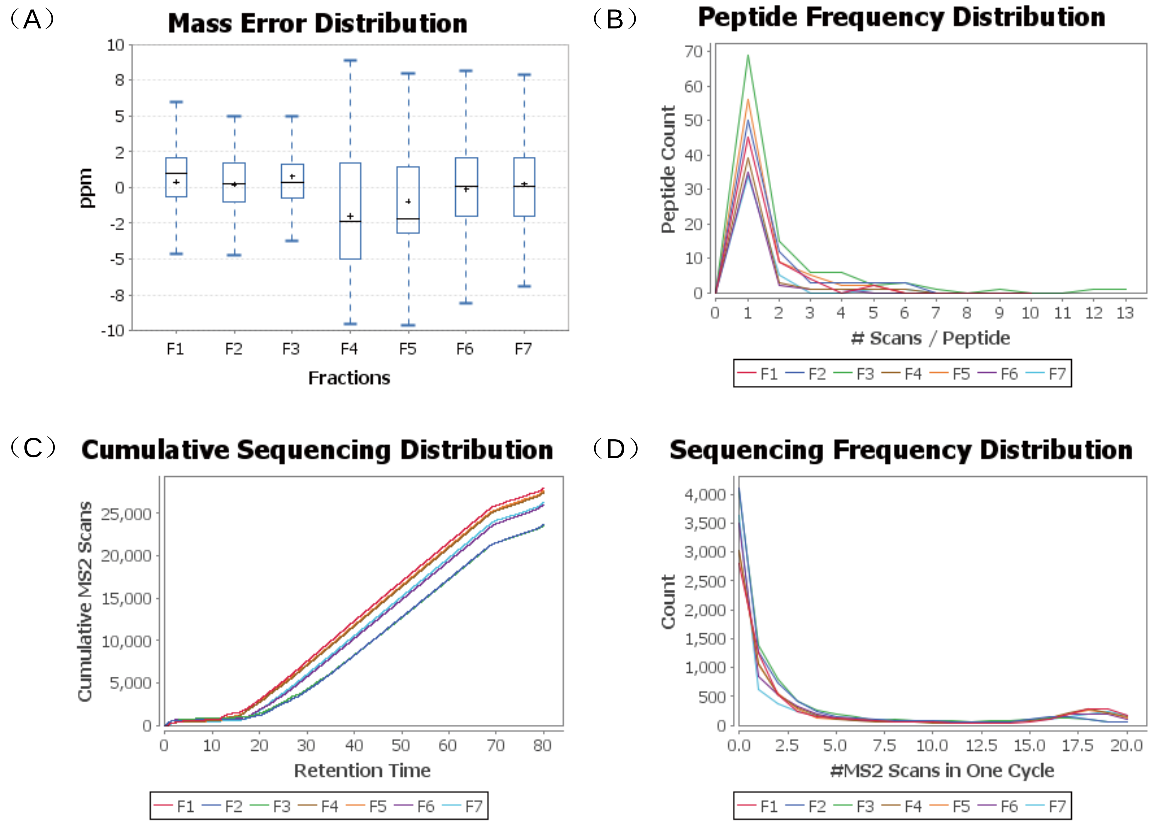


**Figure S1.** Statistics results of mass spectrometry data analysis.(F1: Eurasian elk, F2: Sika deer, F3: Red deer, F4: White-tailed deer, F5: Reindeer, F6: Fallow deer, F7: White-lipped deer)

(A) Precursor mass error for samples: This boxplot shows the precursor mass error for all the samples. (B) Peptide frequency distribution: This graph shows the peptide count in relation to the number of scans per peptide found for each fraction. (C) Cumulative sequencing distribution: This graph shows the acquired cumulative MS2 scans over retention time for each fraction. (D) Sequencing frequency distribution: This graph shows the sequencing count in relation to the number of MS2 scans in one cycle for each fraction.
