# Supplementary material for: Peptide Biomarkers Discovery for Seven Species of Deer Antler Using LC-MS/MS and Label-Free Approach
Source: Molecules. 2022 Jul 25;27(15):4756. doi: 10.3390/molecules27154756 (PMC9331363; doi:10.3390/molecules27154756)
Supplement: Supplementary file 1 [file molecules-27-04756-s001.zip › Table S1.pdf]

Table S1. Deer antler samples used in this study

Code	Species	Origin area	Identification method	Remark
jn011	Sika deer	Jinan, Shandong Prov	DNA barcoding	Analysis and verification
ag013	Sika deer	Anguo, Hebei Prov	DNA barcoding	Verification
ag014	Sika deer	Anguo, Hebei Prov	DNA barcoding	Verification
lk020	Sika deer	Longkou, Shandong Prov	DNA barcoding	Verification
ag017	North American wapiti	Anguo, Hebei Prov	DNA barcoding	Verification
bz001	Red deer	Bozhou, Anhui Prov	DNA barcoding	Analysis and verification
bz002	Red deer	Bozhou, Anhui Prov	DNA barcoding	Verification
bz003	Red deer	Bozhou, Anhui Prov	DNA barcoding	Verification
bz006	Red deer	Bozhou, Anhui Prov	DNA barcoding	Verification
bz007	Red deer	Bozhou, Anhui Prov	DNA barcoding	Verification
bz008	Red deer	Bozhou, Anhui Prov	DNA barcoding	Verification
ag016	Red deer	Anguo, Hebei Prov	DNA barcoding	Verification
43	Reindeer	Xincai, Henan Prov	DNA barcoding	Analysis and verification
Jingxin	Reindeer	Bayannur, Inner Mongolia	Literature method	Verification
10	Reindeer	Bozhou, Anhui Prov	Literature method	Verification
14	Reindeer	Bozhou, Anhui Prov	Literature method	Verification
42	Eurasian elk	Xincai, Henan Prov	DNA barcoding	Analysis and verification
41	Eurasian elk	Xincai, Henan Prov	DNA barcoding	Verification
46	Fallow deer	Xincai, Henan Prov	DNA barcoding	Analysis and verification
47	Fallow deer	Xincai, Henan Prov	DNA barcoding	Verification
21	Fallow deer	Heze, Shandong Prov	DNA barcoding	Verification
45	White-tailed deer	Xincai, Henan Prov	DNA barcoding	Analysis and verification
48	White-lipped deer	Xincai, Henan Prov	DNA barcoding	Analysis and verification
