# Supplementary material for: Peptide Biomarkers Discovery for Seven Species of Deer Antler Using LC-MS/MS and Label-Free Approach
Source: Molecules. 2022 Jul 25;27(15):4756. doi: 10.3390/molecules27154756 (PMC9331363; doi:10.3390/molecules27154756)
Supplement: Supplementary file 1 [file molecules-27-04756-s001.zip › Table S2.pdf]

Table S2. Database parameters of "adult beta-globin of deer"

Entry	Entry name	Protein names	Organism	URL
A0A220IG96	A0A220IG96_ELADA	Adult beta globin	Pere David's deer (*Elaphurus davidianus*)	https://www.uniprot.org/uniprot/A0A220IG96
A0A220IG99	A0A220IG99_DAMDA	Adult beta globin	Fallow deer (*Dama. dama*)	https://www.uniprot.org/uniprot/A0A220IG99
A0A220IGA2	A0A220IGA2_PRZAL	Adult beta globin	White-lipped deer (*Przewalskium. albirostris*)	https://www.uniprot.org/uniprot/A0A220IGA2
A0A220IGA1	A0A220IGA1_RUCDU	Adult beta globin	Cervus duvaucelii (*Rucervus duvaucelii*)	https://www.uniprot.org/uniprot/A0A220IGA1
A0A220IG98	A0A220IG98_9CETA	Adult beta globin	Bactrian deer (*Cervus hanglu bactrianus*)	https://www.uniprot.org/uniprot/A0A220IG98
A0A220IG91	A0A220IG91_ODOVR	Adult beta globin	White-tailed deer (*Odocoileus virginianus*)	https://www.uniprot.org/uniprot/A0A220IG91
A0A220IGA7	A0A220IGA7_HYDIN	Adult beta globin	Chinese water deer (*Hydropotes inermis*)	https://www.uniprot.org/uniprot/A0A220IGA7
A0A220IGB0	A0A220IGB0_CAPCA	Adult beta globin	European roe deer (*Capreolus capreolus*)	https://www.uniprot.org/uniprot/A0A220IGB0
A0A220IG97	A0A220IG97_CERNI	Adult beta globin	Sika deer (*Cervus. nippon*)	https://www.uniprot.org/uniprot/A0A220IG97
A0A220IGB1	A0A220IGB1_CEREL	Adult beta globin	Red deer (*Cervus. elaphus*)	https://www.uniprot.org/uniprot/A0A220IGB1
A0A220IG90	A0A220IG90_9CETA	Adult beta globin	Eurasian elk (*Alces. alces*)	https://www.uniprot.org/uniprot/A0A220IG90
A0A220IG93	A0A220IG93_PUDPU	Adult beta globin	Southern pudu (*Pudu pudu*)	https://www.uniprot.org/uniprot/A0A220IG93
A0A220IGA3	A0A220IGA3_9CETA	Adult beta globin	North American wapiti (*Cervus. canadensis*)	https://www.uniprot.org/uniprot/A0A220IGA3
A0A220IG95	A0A220IG95_MUNRE	Adult beta globin	Reeves' muntjac (*Muntiacus reevesi*)	https://www.uniprot.org/uniprot/A0A220IG95
A0A220IGA4	A0A220IGA4_RANTA	Adult beta globin	Reindeer (*Rangifer. tarandus*)	https://www.uniprot.org/uniprot/A0A220IGA4
