# Supplementary material for: Peptide Biomarkers Discovery for Seven Species of Deer Antler Using LC-MS/MS and Label-Free Approach
Source: Molecules. 2022 Jul 25;27(15):4756. doi: 10.3390/molecules27154756 (PMC9331363; doi:10.3390/molecules27154756)
Supplement: Supplementary file 1 [file molecules-27-04756-s001.zip › Table S3.pdf]

Table S3. Statistics results of mass spectrometry data analysis

Analysis type	Analyzed items	Total	Average	Control: Eurasian elk	Sika deer	Red deer	White-tailed deer	Reindeer	Fallow deer	White-lipped deer
Chromatography Analysis	Features	196343	28049	25837	32473	31587	29048	28629	25555	23214
	Full Width (min)	0.19	0.19	0.24	0.18	0.2	0.2	0.2	0.17	0.16
	Full Width at Half Maximum (sec)	6.75	6.77	8.93	6.32	6.7	6.99	7.07	5.91	5.49
Acquisition Analysis	MS1	50610	7230	6638	8297	8468	6740	6708	7020	6739
	MS/MS	182536	26077	27988	23658	23531	27460	27647	26007	26245
	MS2/MS1 Rate	3.61	3.66	4.22	2.85	2.78	4.07	4.12	3.7	3.89
Identification Result	Peptide Spectrum Matches	694	99	85	128	210	63	107	46	55
	MS/MS Scans	694	99	85	128	210	63	107	46	55
	Identified Features	481	69	51	110	163	30	70	21	36
	Peptides	333	71	63	93	123	52	82	42	45
	Sequences	258	63	60	74	105	46	74	39	41
	All Proteins	24	24	24	24	24	24	24	24	24
	ID Rate	0.38%	0.39%	0.30%	0.54%	0.89%	0.23%	0.39%	0.18%	0.21%
	Scans Peptides Rate	2.08	1.32	1.35	1.38	1.71	1.21	1.3	1.1	1.22
